# Negative and Positive Association Rules Mining from Text Using Frequent and Infrequent Itemsets

**DOI:** 10.1155/2014/973750

**Published:** 2014-05-18

**Authors:** Sajid Mahmood, Muhammad Shahbaz, Aziz Guergachi

**Affiliations:** ^1^Department of Computer Science & Engineering, University of Engineering & Technology, Lahore, Pakistan; ^2^Al-Khawarizmi Institute of Computer Sciences, UET, Lahore, Pakistan; ^3^Ted Rogers School of Information Technology Management, Ryerson University, Toronto, Canada

## Abstract

Association rule mining research typically focuses on positive association rules (PARs), generated from frequently occurring itemsets. However, in recent years, there has been a significant research focused on finding interesting infrequent itemsets leading to the discovery of negative association rules (NARs). The discovery of infrequent itemsets is far more difficult than their counterparts, that is, frequent itemsets. These problems include infrequent itemsets discovery and generation of accurate NARs, and their huge number as compared with positive association rules. In medical science, for example, one is interested in factors which can either adjudicate the presence of a disease or write-off of its possibility. The vivid positive symptoms are often obvious; however, negative symptoms are subtler and more difficult to recognize and diagnose. In this paper, we propose an algorithm for discovering positive and negative association rules among frequent and infrequent itemsets. We identify associations among medications, symptoms, and laboratory results using state-of-the-art data mining technology.

## 1. Introduction

Association rules (ARs), a branch of data mining, have been studied successfully and extensively in many application domains including market basket analysis, intrusion detection, diagnosis decisions support, and telecommunications. However, the discovery of associations in an efficient way has been a major focus of the data mining research community [1–6].

Traditionally, the association rule mining algorithms target the extraction of frequent features (itemsets), that is, features boasting high frequency in a transactional database. However, many important itemsets, with low support (i.e., infrequent), are ignored by these algorithms. These infrequent itemsets, despite their low support, can produce potentially important negative association rules (NARs) with high confidences, which are not observable among frequent data items. Therefore, discovery of potential negative association rules is important to build a reliable decision support system. The research in this paper extends discovery of positive as well as negative association rules of the forms *A*⇒¬*B*, ¬*A*⇒*B*, ¬*A*⇒¬*B*, and so forth.

The number of people discussing their health in blogs and other online discussion forums is growing rapidly [7, 8]. Patient-authored blogs have now become an important component of modern-day healthcare. These blogs can be effectively used for decision support and quality assurance. Patient-authored blogs, where patients give an account of their personal experiences, offer near accurate and complete problem lists with symptoms and ongoing treatments [9]. In this paper, we have investigated the efficient mechanism of identifying positive and negative associations among medications, symptoms, and laboratory results using state-of-the-art data mining technology. Rules of the form *A*⇒*B* or *A*⇒¬*B* can help explain the presence or absence of different factors/variables. Such types of associations can be useful for building decision support systems in the healthcare sector.

We target 3 major problems in association rule mining: (a) effectively extracting positive and negative association rules from text datasets, (b) extracting negative association rules from the frequent itemsets, and (c) the extraction of positive association rules from infrequent itemsets.

The rest of this paper is organized as follows. In the next section, we present brief introduction to data mining terminology and background.  Section 3 reviews related work on association rule mining. In Section 4, we describe the methodology for identifying both frequent and infrequent itemsets of interest and generation of association rules based on these itemsets. The proposed model for extracting positive and negative association rules is presented in Section 5 and the final section of this paper gives the details of the experimental results, comparisons, and conclusions.  Section 6 presents the experimental results and the conclusion and future directions are given in Section 7.

## 2. Terminology and Background

Let us consider *I* = {*i*_1_, *i*_2_,…, *i*_*N*_} as a set of *N* distinct literals/terms called* items* and let *D* be a database of transactions (documents/blogs, etc.), where each transaction *T* is a set of items/terms such that *T* is a subset of “*I*.” Each transaction is associated with a unique identifier, called *T*_*ID*_. Let *A*, *B* be sets of items; an association rule is a derivation of the form *A*⇒*B*, where *A* ⊂ *I*, *B* ⊂ *I*, and *A*∩*B* = *Ø*. “*A*” is called the antecedent of the rule, and “*B*” is called the consequent of the rule. An association rule *A*⇒*B* can have different measures denoting its significance and quality. In our approach, we have employed (i) support, by denoting it by supp which is the percentage of transactions in database *D* containing both *A* and *B*, (ii) confidence, by denoting it by conf which is representing the percentage of transactions in *D* containing *A* that also contain *B* which can be denoted in probability terms by *P*(*B* | *A*), and (iii) lift, by denoting it by lift characterizing the direction of relationship between the antecedent and consequent of the association rule. Rules having the support value greater than user defined minimum support minsupp, in which the itemset needs to be present in minimum threshold number of transactions, and confidence greater than user defined minimum confidence minconf are called valid association rules. The lift symbolizes the association whether positive or negative. A value of lift greater than 1 indicates a positive relationship between the itemsets; value of lift less than 1 indicates a negative relationship; and where the value of lift equals 1, the itemsets are independent and there exists no relationship between the itemsets.

Some of the above and derived definitions can be represented with the following mathematical equations:

(1) supp⁡(A) /∗Number/Percentage  of  transaction(s)containing  A/∗

(2) supp⁡(A⟹B) =P(AB) /∗Number/Percentage  of   transactions  where  A  and  B  co-exist/∗

(3) conf(A⟹B) =P(AB)P(A) /∗Confidence  measure  of  the  rule  thatwhenever  A  occurs  B  also  occursin  transaction(s)/∗

(4) lift(A⟹B) =P(AB)P(A)P(B) /∗The  strength  of  relationshipbetween  A  and  B/∗ =1+P(AB)−P(A)P(B)P(A)P(B)

(5) $$\begin{array}{c} \text{Supp}\left(\neg A\right)=1-\text{Supp}\left(A\right) \end{array}$$

(6) $$\begin{array}{c} \text{Supp}\left(A\cup \neg B\right)=\text{Supp}\left(A\right)-\text{Supp}\left(A\cup B\right) \end{array}$$

(7) $$\begin{array}{c} \text{Conf}\left(A⟹\neg B\right)=1-\text{Conf}\left(A⟹B\right)=\frac{P\left(A\neg B\right)}{P\left(A\right)} \end{array}$$

(8) $$\begin{array}{c} \text{Supp}\left(\neg A\cup B\right)=\text{Supp}\left(B\right)-\text{Supp}\left(B\cup A\right) \end{array}$$

(9) Conf(¬A⟹B)=Supp(B)(1−Conf(B⟹A))1−P(A)=Supp(¬A∪B)Supp(¬A)

(10) $$\begin{array}{c} \text{Supp}\left(\neg A\cup \neg B\right)=1-\text{Supp}\left(A\right)-\text{Supp}\left(B\right)+\text{Supp}\left(A\cup B\right) \end{array}$$

(11) Conf(¬A⟹¬B) =1−Supp(A)−Supp(B)+Supp(A∪B)1−P(A) =Supp(¬A∪¬B)Supp(¬A).

The huge number of infrequent items generates an even higher number of negative rules in comparison to the positive association rules. The problem is overwhelmed when dealing with text where words/terms are items and the documents are transactions. It is also difficult to set a threshold for the minimum support as a measure for text because of the huge number of unique and sporadic items (words) in a textual dataset. Indexing (assigning weights to the terms) of the text documents is very important for them to be used as transactions for extracting association rules. Indexing techniques from the information retrieval field [10] can greatly benefit in this regard. Index terms, the words whose semantics help in identifying the document's main subject matter [11], help describe a document. They possess different relevance to a given document in the collection and, therefore, the assigned different numerical weights. Text mining aims to retrieve information from unstructured text to present the extracted knowledge in a compact form to the users [12]. The primary goal is to provide the users with knowledge for the research and educational purposes.

It is, therefore, imperative to employ some weight assignment mechanism. We have used inverse document frequency (IDF), which denotes the importance of a term in a corpus. Selection of features on the basis of IDF values needs a careful reading, that is, which range of IDF value features is included. This can greatly affect the results as choosing a very low value of “*N*” is feared to nullify the impact of IDF, and choosing a very high value may result in losing important terms/features of the dataset. We have proposed a user tuned parameter, that is, top *N*%, where the value of “*N*” is chosen by the user, depending upon the characteristics of the data.

### 2.1. IDF

IDF weighting scheme is based on the intuition of term occurrence in a corpus. It surmises that the fewer the documents containing a term are, the more discerning they become and hence an important artefact. The IDF helps in understanding the terms that carry special significance in a certain type of document corpus. It assigns high weight to terms that occur rarely in a document corpus. In a medical corpus, for instance, the word “disease” is not likely to carry a significant meaning. Instead a disease name, for example, “cholera” or “cancer,” would carry significant meanings in characterizing the document.

Keeping this in view, a higher weight should be assigned to the words that appear in documents in close connection with a certain topic, while a lower weight should be assigned to those words that show up without any contextual background. IDF weighting is a broadly used method for text analysis [10, 13]. We can mathematically represent IDF as IDF_*t*_ = log⁡⁡(*D*/*df*_*t*_), where *t* is the term whose weight is to be calculated, *df*_*t*_ represents the documents in which the term is present, and *D* symbolizes the document corpus.

Table 1 shows the example set of words and their respective IDF scores in a document corpus with a threshold score value of 60%.

The number of words in the documents before IDF score calculation and top 60% selection is 280254 and, after selecting the top 60% based on IDF scores, goes down to 81733. The bold part of the table shows a sample of the eradicated words which have IDF scores below threshold value.

## 3. Literature Review

The association rule mining is aimed at the discovery of associations among itemsets of a transactional database. Researchers have extensively studied association rules since their introduction by [14]. Apriori is the most well-known association rule mining algorithm. The algorithm has a two-step process: (1) frequent itemset generation (doing multiple scans of the database) and (2) association rule generation. The major advantage of Apriori over other association rule mining algorithms is its simple and easy implementation. However, multiple scans over the database to find rules make Apriori algorithm's convergence slower for large databases.

The other popular association rule mining algorithm, frequent pattern-growth (FP-Growth), proposed by Han et al. [3], compresses the data into an FP-Tree for identifying frequent itemsets. FP algorithm makes fewer scans of the database, making it practically usable for large databases like text.

However, there has been very little research done for finding negative association rules among infrequent itemsets. The association rule mining algorithms are seldom designed for mining negative association rules. Most of the existing algorithms can rarely be applied in their current capacity in the context of negative association rule mining. The recent past, however, has witnessed a shift in the focus of the association rule mining community, which is now focusing more on negative association rules extraction [1, 15–19]. Delgado et al. have proposed a framework for fuzzy rules that extends the interesting measures for their validation from the crisp to the fuzzy case [20]. A fuzzy approach for mining association rules using the crisp methodology that involves the absent items is presented in [21].

Savasere et al. [22] presented an algorithm for extracting strong negative association rules. They combine frequent itemsets and the domain knowledge, to form taxonomy, for mining negative association rules. Their approach, however, requires users to set a predefined domain dependent hierarchical classification structure, which makes it difficult and hard to generalize. Morzy presented the DI-Apriori algorithm for extracting dissociation rules among negatively associated itemsets. The algorithm keeps the number of generated patterns low; however, the proposed method does not capture all types of generalized association rules [23].

Dong et al. [16] introduced an interest value for generating both positive and negative candidate items. The algorithm is Apriori-like for mining positive and negative association rules. Antonie and Zaïane [15] presented a {*support*, *confidence* *and* *correlation*} coefficient based algorithm for mining positive and negative association rules; the coefficients need to be continuously updated while running the algorithm; also, the generation of all negative association rules is not guaranteed.

Wu et al. proposed negative and positive rule mining framework, based on the Apriori algorithm. Their work does not focus on itemset dependency; rather, it focuses on rule dependency measures. More specifically, they have employed the Piatetsky-Shapiro argument [24] about the association rules; that is, a rule *X*⇒*Y* is interesting if and only if supp⁡(*A*⇒*B*) ≠ supp⁡(*A*)supp⁡(*B*). This can be further explained using the following.

A rule is as follows: *A*⇒¬*B* is valid if and only if

1. lift(*A*, ¬*B*) = |supp⁡(*A* ∪ ¬*B*) − supp⁡(*A*)supp⁡(¬*B*)| > 1 indicates a positive relationship between *X* and ¬*Y*,
2. the other condition for a rule to be valid implies that {*X*}, {*Y*} *ϵ* frequent  itemsets.

The second condition can greatly increase the efficiency of the rule mining process because all the antecedent or consequent parts of ARs can be ignored where {*X*}, {*Y*} *ϵ* Infrequent  itemset. This is needed because the itemset is infrequent so Apriori does not guarantee the subsets to be frequent.

Association rule mining research mostly concentrates on positive association rules. The negative association rule mining methods reported in literature generally target market basket data or other numeric or structured data. Complexity of generating negative association rules from text data thus becomes twofold, that is, dealing with the text and generating negative association rules as well as positive rules. As we demonstrate in the later sections, negative association rule mining from text is different from discovering association rules in numeric databases, and identifying negative associations raises new problems such as dealing with frequent itemsets of interest and the number of involved infrequent itemsets. This necessitates the exploration of specific and efficient mining models for discovering positive and negative association rules from text databases.

## 4. Extraction of Association Rules

We present an algorithm for mining both positive and negative association rules from frequent and infrequent itemsets. The algorithm discovers a complete set of positive and negative association rules simultaneously. There are few algorithms for mining association rules for both frequent and infrequent itemsets from textual datasets.

We can divide association rule mining into the following:

1. finding the interesting frequent and infrequent itemsets in the database *D*,
2. finding positive and negative association rules from the frequent and infrequent itemsets, which we get in the first step.

The mining of association rules appears to be the core issue; however, generation and selection of interesting frequent and infrequent itemsets is equally important. We discuss the details of both in the following discussion.

### 4.1. Identifying Frequent and Infrequent Itemsets

As mentioned above, the number of extracted items (both frequent and infrequent) from the text datasets can be very large, with only a fraction of them being important enough for generating the interesting association rules. Selection of the useful itemsets, therefore, is challenging. The support of an item is a relative measure, with respect to the database/corpus size. Let us suppose that the support of an itemset *X* is 0.4 in 100 transactions; that is, 40% of transactions contain the itemset. Now, if 100 more transactions are added to the dataset, and only 10% of the added 100 transactions contain the itemset *X*, the overall support of the itemset *X* will be 0.25; that is, 25% of the transactions now contain itemset *X*. Therefore, the support of an itemset is a relative measure. Hence, we cannot rely only on support measure for the selection of important/frequent itemsets.

The handling of large number of itemsets, which is the case when dealing with textual datasets, is more evident when dealing with infrequent itemsets. This is because the number of infrequent itemsets rises exponentially [1]. Therefore, we only select those terms/items from the collection of documents, which have importance in the corpus. This is done using the IDF weight assigning method. We filter out words either not occurring frequently enough or having a near constant distribution among the different documents. We use top-*N*% age (for a user specified *N*, we used 60%) as the final set of keywords to be used in the text mining phase [25]. The keywords are sorted in descending order by the algorithm based on their IDF scores.

### 4.2. Identifying Valid Positive and Negative Association Rules

By a valid association rule, we mean any expression of the form *A*⇒*B*, where *A* ∈ {*X*, ¬*X*}, *B* ∈ {*Y*, ¬*Y*}, *X*, *Y* ⊂ *I*, and *X*∩*Y* = *∅*, s.t. Consider

- supp⁡(*A*⇒*B*) ≥ ms,
- supp⁡(*X*) ≥ ms, supp⁡(*Y*) ≥ ms,
- conf(*A*⇒*B*) ≥ mc,
- lift(*A*⇒*B*) > 1.

Let us consider an example, from our medical “cancer” blogs' text dataset. We analyze people's behavior about the cancer and mole. We have

- supp⁡(mole) = 0.4, supp⁡(¬mole) = 0.6,
- supp⁡(cancer) = 0.6, supp⁡(¬cancer) = 0.4,
- supp⁡(cancer ∪ mole) = 0.05,
- min_supp = 0.2,
- min_conf = 0.6.

From above we can see the following:

- supp⁡(cancer ∪ mole) = 0.05 < min_supp; therefore, {mole ∪ cancer} is an infrequent itemset (inFIS);
- conf(mole⇒cancer) = 0.125 < min_conf; from (3),
  - therefore, (mole⇒cancer) cannot be generated as a valid rule using the support-confidence framework.

Now, we try to generate a negative rule from this example:

- supp⁡(mole ∪ ¬cancer) = supp⁡(mole) − supp⁡(cancer ∪ mole):
  - = 0.4 − 0.05 = 0.35 > min_supp, from (6);
- conf(mole⇒¬cancer) = supp⁡(mole ∪ ¬cancer)/supp⁡(mole), from (7):
  - = 0.35/0.4 = 0.875 > min_conf; therefore, we can generate (mole⇒¬cancer) as a negative rule;
- lift(mole⇒¬cancer) = supp⁡(mole ∪ ¬cancer)/supp⁡(mole)supp⁡(¬cancer):
  - = 0.35/(0.4∗0.4) = 2.1875 > 1, which is much greater than 1 showing a strong relation between the presence of mole and absence of cancer; therefore, we can generate (mole⇒¬cancer) as a valid negative rule with 87.5% confidence and strong association relationship between the presence and absence of mole and cancer, respectively.

The above example clearly shows the importance of infrequent itemsets and the generated negative association rules and their capability to track the important implications/associations, which would have been missed when mining only positive association rules.

### 4.3. Proposed Algorithm: Apriori FISinFIS (See Algorithm 1)

The Apriori FISinFIS procedure generates all frequent and infrequent itemsets of interest in a given database *D*, where FIS is the set of all frequent itemsets of interest in *D* and inFIS is the set of all infrequent itemsets in *D*. FIS and inFIS contain only frequent and infrequent itemsets of interest, respectively.

The initialization is done in Step (1). Step (2) generates temp_1_, all itemsets of size 1; in step (2.1), we generate FIS_1_, all frequent itemsets of size 1, while, in step (2.2), all infrequent itemsets of size 1 in database *D* are generated in inFIS_1_ in the first pass of *D*.

Step (3) generates FIS_*k*_ and inFIS_*k*_ for *k* ≥ 2 by a loop, where FIS_*k*_ is the set of all frequent *k*-itemsets, which have greater support than user defined minimum threshold, in the *k*th pass of *D*; inFIS_*k*_ is the set of all infrequent *k*-itemsets, which have less support than user defined minimum threshold. The loop terminates when all the temporary itemsets have been tried; that is, temp_*k*−1_ = *∅*. For each pass of the database in Step (3), say pass *k*, there are five substeps as follows.

Step (3.1) generates candidate itemsets *C*_*k*_ of all *k*-itemsets in *D*, where each *k*-itemset in *C*_*k*_ is generated by two frequent itemsets in temp_*k*−1_. The itemsets in *C*_*k*_ are counted in *D* using a loop in Step (3.2). Step (3.3) calculates support of each itemset in *C*_*k*_ and step (3.4) stores the generated itemsets in a temporary data structure. We have used an implementation of “*HashMap*” in our experimentation as a temporary data structure.

Then FIS_*k*_ and inFIS_*k*_ are generated in Steps (4) and (5), respectively. FIS_*k*_ is the set of all potentially useful frequent *k*-itemsets in temp_*k*_, which have greater support value than the minsupp. inFIS_*k*_ is the set of all infrequent *k*-itemsets in temp_*k*_, which have less support values than the minsupp. The FIS_*k*_ and inFIS_*k*_ are added to the FIS and inFIS in Steps (6) and (7). Step (8) increments the itemset size. The procedure ends in Step (9) which outputs frequent and infrequent itemsets in FIS and inFIS, respectively.

### 4.4. Algorithm: FISinFIS Based Positive and Negative Association Rule Mining

Algorithm 2 generates positive and negative association rules from both the frequent itemsets (FIS) and infrequent itemsets (inFIS). Step (1) initializes the positive and negative association rule sets as empty. Step (2) generates association rules from FIS; in step (2.1), positive association rules of the form *A*⇒*B* or *B*⇒*A*, which have greater confidence than the user defined threshold and lift greater than 1, are extracted as valid positive association rules. Step (2.2) generates negative association rules of the form *A*⇒¬*B*, ¬*A*⇒*B*, ¬*A*⇒¬*B*, and so forth, which have greater confidence than the user defined threshold and lift greater than 1, are extracted as valid negative association rules (Figure 2).

Step (3) generates association rules from inFIS; in step (3.1), positive association rule of the form *A*⇒*B* or *B*⇒*A*, which has greater confidence than the user defined threshold and lift greater than 1, is extracted as a valid positive association rule. Step (3.2) generates negative association rule of the form *A*⇒¬*B*, ¬*A*⇒*B*, or ¬*A*⇒¬*B*, which has greater confidence than the user defined threshold and lift greater than 1, and is extracted as a valid negative association rule.

## 5. Discovering Association Rules among Frequent and Infrequent Items

Mining positive association rules from frequent itemsets is relatively a trivial issue and has been extensively studied in the literature. Mining negative association rules of the form *A*⇒¬*B*, ¬*A*⇒*B*, *B*⇒¬*A*, or ¬*B*⇒*A*, and so forth from textual datasets, however, is a difficult task, where *A* ∪ *B* is a nonfrequent itemset. Database (corpus) *D* has an exponential score of nonfrequent itemsets; therefore, negative association rule mining stipulates the examination of much more search space than positive association rules.

We, in this work, propose that, for the itemsets occurring frequently, given the user defined min-sup, their subitems can be negatively correlated leading to the discovery of negative association rules. Similarly, the infrequent itemsets may have their subitems with a strong positive correlation leading to the discovery of positive association rules.

Let *I* be the set of items in database *D*, such that

 
*I* = *A* ∪ *B*,
 
*A*∩*B* = *φ*.

Thresholds minsup and minconf are given by the user.

### 5.1. Generating Association Rules among Frequent Itemsets

See Algorithm 3.

### 5.2. Generating Association Rules among Infrequent Itemsets

For brevity, we only consider one form of association from both positive and negative; that is, *A*⇒*B* and *A*⇒¬*B*; the other forms can similarly be extracted.

In the description of association rules as shown in Algorithm 4, supp⁡(*A* ∪ *B*) ≥ minsupp guarantees that the association rule describes the relationship among items of a frequent itemset, whereas supp⁡(*A* ∪ *B*) < minsupp guarantees that the association rule describes the relationship among items of an infrequent itemset; however, the subitems of the itemset need to be frequent as enforced by the conditions supp⁡(*A*) ≥ minsupp and supp⁡(*B*) ≥ minsupp. The interestingness measure, lift, has to be greater than 1, articulating a positive dependency among the itemsets; the value of lift less than 1 will articulate a negative relationship among the itemsets.

The algorithm generates a complete set of positive and negative association rules from both frequent and infrequent itemsets. The frequent itemsets have traditionally been used to generate positive association rules; however, we argue that items in frequent itemsets can be negatively correlated. This can be illustrated using the following example:

 minsupp = 0.2, minconf = 0.6;
 supp⁡(*A*) = 0.7, supp⁡(*B*) = 0.5, supp⁡(*A* ∪ *B*) = 0.25;
 conf(*A*⇒*B*) = 0.3571, lift(*A*⇒*B*) = 0.7143;
 conf(*B*⇒*A*) = 0.5, lift(*B*⇒*A*) = 0.7143;
 supp⁡(*A* ∪ ¬*B*) = 0.7 − 0.25 = 0.45, conf(*A*⇒¬*B*) = 0.45/0.7 = 0.6429;
 lift(*A*⇒¬*B*) = 0.45/(0.7∗0.5) = 1.2857.

The above example clearly shows that itemsets, despite being frequent, can have negative relationships among their item subsets. Therefore, in Step (2.2) of Algorithm 2, we try to generate negative association rules using frequent itemsets.

The infrequent itemsets, on the other hand, have either been totally ignored while generating associations or mostly used to generate only negative association rules. However, infrequent itemsets have potentially valid and important positive association rules among them having high confidence and strongly positive correlation. This can be illustrated using the following example:

 minsupp = 0.3, minconf = 0.7;
 supp⁡(*A*) = 0.8, supp⁡(*B*) = 0.2, supp⁡(*A* ∪ *B*) = 0.2;
 conf(*A*⇒*B*) = 0.25, lift(*A*⇒*B*) = 1.19;
 conf(*B*⇒*A*) = 1, lift(*B*⇒*A*) = 1.19.

We can visualize from the example that (*A* ∪ *B*), in spite of being an infrequent itemset, has a very strong positive association *B*⇒*A* having 100% confidence and a positive correlation. Our proposed algorithm covers the generation of such rules as explained in Step (3.1) of our proposed algorithm.

## 6. Experimental Results and Discussions

We performed our experiments on medical blogs datasets, mostly authored by patients writing about their problems and experiences. The datasets have been collected from different medical blog sites:

- Cancer Survivors Network [http://csn.cancer.org/],
- Care Pages [http://www.carepages.com/forums/cancer].

The blogs text was preprocessed before the experimentation, that is, stop words removal, stemming/lemmatization, nonmedical words removal, and so forth. We assigned weights to terms/items after preprocessing using the IDF scheme, for selecting only the important and relevant terms/items in the dataset. The main parameters of the databases are as follows:

- the total number of blogs (i.e., transactions) used in this experimentation was 1926;
- the average number of words (attributes) per blog (transaction) was 145;
- the smallest blog contained 79 words;
- the largest blog contained 376 words;
- the total number of words (i.e., attributes) was 280254 without stop words removal;
- the total number of words (i.e., attributes) was 192738 after stop words removal;
- the total number of words selected using top-*N*% age of IDF words was 81733;
- algorithm is implemented in java.

**Figure 1:**
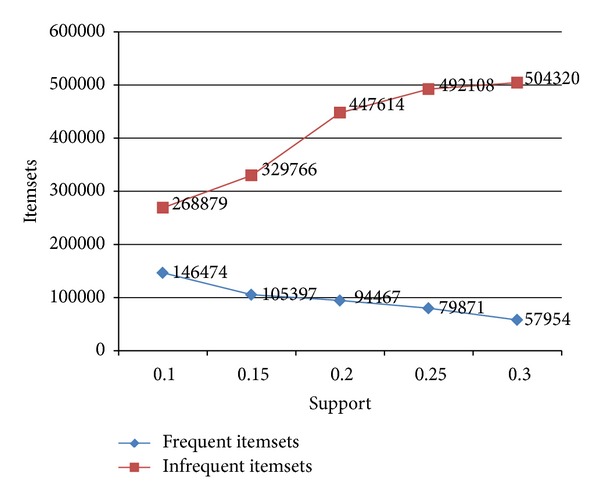
Frequent and infrequent itemsets generated with varying minimum support values.

**Figure 2:**
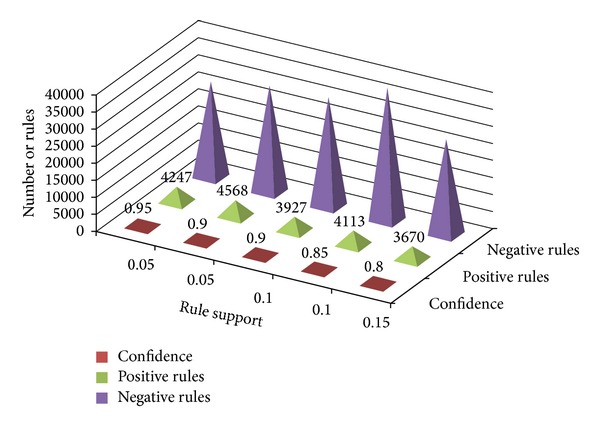
Positive and negative rules generated with varying minimum supports and confidence values.

Table 2 summarizes the number of itemsets generated with varying minsup values. We can see that the number of frequent itemsets decreases as we increase the minsup value. However, a sharp increase in the number of infrequent itemsets can be observed. This can also be visualized in Figure 1.

Table 3 gives an account of the experimental results for different values of minimum support and minimum confidence. The lift value has to be greater than 1 for a positive relationship between the itemsets; the resulting rule, however, may itself be positive or negative. The total number of positive rules and negative rules generated from both frequent and infrequent itemsets is given.

Although the experimental results greatly depend on the datasets used, they still flaunt the importance of IDF factor in selecting the frequent itemsets, along with the generation of* negative rules from frequent itemsets* and the extraction of* positive rules from infrequent itemsets*. The number of negative rules generated greatly outnumbers the positive rules not only because of the much more infrequent itemsets as compared to frequent itemsets but also because of finding the negative correlation between the frequent itemsets, using proposed approach, leading to the generation of negative association rules.

The frequent and infrequent itemset generation using Apriori algorithm takes only a little extra time as compared to the traditional frequent itemset finding using Apriori algorithm. This is because each item's support is calculated for checking against the threshold support value to be classified as frequent and infrequent; therefore, we get the infrequent items in the same pass as we get frequent items. However, the processing of frequent and infrequent itemsets for the generation of association rules is different. For frequent items generated through Apriori algorithm, they have an inherent property that their subsets are also frequent; however, we cannot guarantee that for the infrequent itemsets. Thus, we impose an additional check on the infrequent itemsets that their subsets are frequent when generating association rules among them.

The researches on mining association rules among the frequent and infrequent itemsets have been far and few, especially from the textual datasets. We have proposed this algorithm which can extract both types of association rules, that is, positive and negative, among both frequent and infrequent itemsets. We give a sample of all four types of association rules extracted using the algorithm.

Table 4 gives a summary of the generated association rules. The four (4) types of generated association rules are illustrated. Table 4(a) shows a sample of positive association rules generated from the frequent itemsets. Table 4(b) shows negative association rules generated from the frequent itemsets. This has not yet been explored by the research community. Sample of positive association rules generated from the infrequent itemsets are demonstrated in Table 4(c). This type of association rules would potentially be useful and researchers are interested to extract them. There is no research done in this domain of extracting positive association rules from infrequent itemsets in the textual data before this research. Table 4(d) shows the results of negative association rules from the infrequent itemsets.

## 7. Concluding Remarks and Future Work

Identification of associations among symptoms and diseases is important in diagnosis. The field of negative association rules (NARs) mining holds enormous potential to help medical practitioners in this regard. Both the positive and negative association rule mining (PNARM) can hugely benefit the medical domain. Positive and negative associations among diseases, symptoms, and laboratory test results can help a medical practitioner reach a conclusion about the presence or absence of a possible disease. There is a need to minimize the errors in diagnosis and maximize the possibility of early disease identification by developing a decision support system that takes advantage of the NARs. Positive association rules such as *Flu*⇒*Headache* can tell us that* Headache* is experienced by a person who is suffering from* Flu*. On the contrary, negative association rules such as ¬*Throbbing*-*Headache*⇒¬*Migraine* tell us that if* Headache* experienced by a person is not* Throbbing*, then he may not have* Migraine* with a certain degree of confidence. The applications of this work include the development of medical decision support system, among others, by finding associations and dissociations among diseases, symptoms, and other health-related terminologies. The current algorithm does not account for the context and semantics of the terms/items in the textual data. In future, we plan to assimilate the context of the features in our work, in order to perk up the quality and efficacy of the generated association rules.

In this paper, contributions to the NARM research were made by proposing an algorithm for efficiently generating negative association rules, along with the positive association rules. We have proposed a novel method that captures the negative associations among frequent itemsets and also extracts the positive associations among the infrequent itemsets. Whereas, traditional association rule mining algorithms have focused on frequent items for generating positive association rules and have used the infrequent items for the generation of negative association rules. The experimental results have demonstrated that the proposed approach is effective, efficient and promising.

## Figures and Tables

**Algorithm 1:**
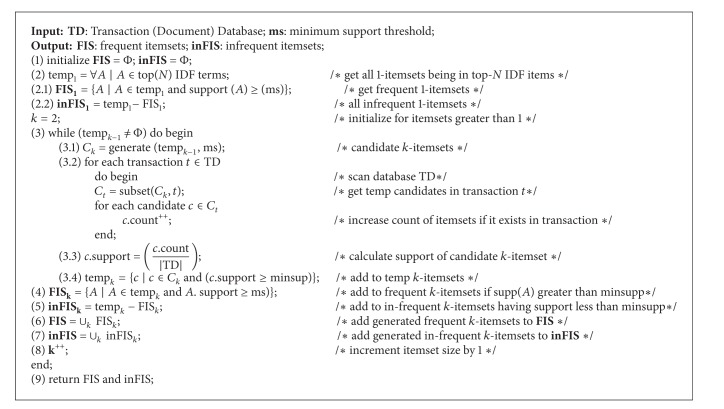


**Algorithm 2:**
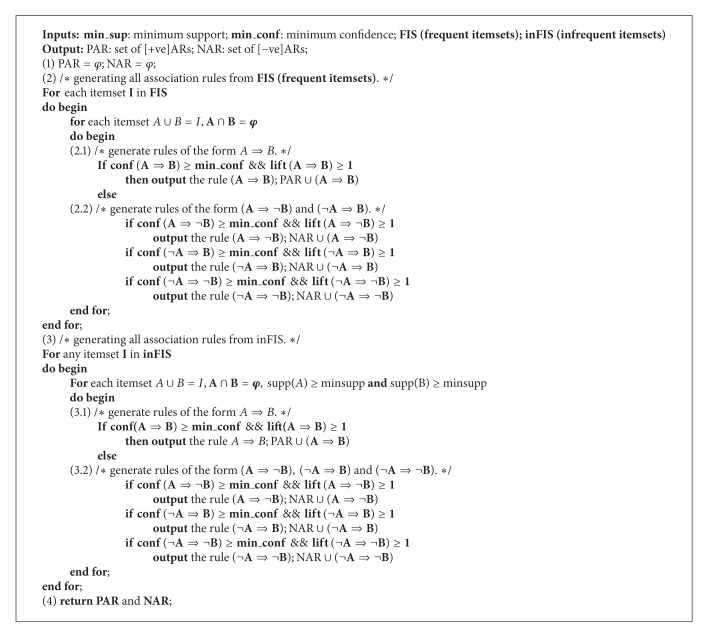


**Algorithm 3:**
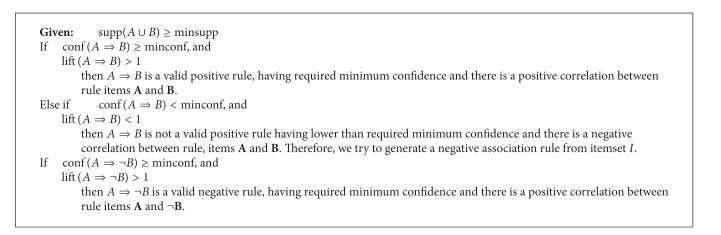


**Algorithm 4:**
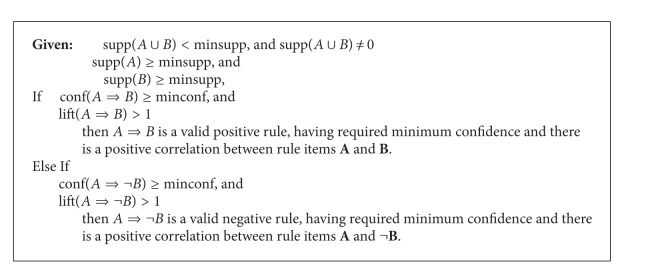


**Table 1:** IDF scores of sample keywords of the corpus.

Selected keywords	IDF	**Discarded keywords**	**IDF**
Chemo	2.2693	**Threat**	**0.0158**
Radiation	2.2535	**Disease**	**0.0157**
Tumor	2.2316	**Severe**	**0.01426**
Surgery	2.2135	**Produce**	**0.01392**
Cancer	2.2013	**Result**	**0.01194**
Temodar	1.9830	**Need**	**0.00639**
CT scan	1.9812	**Analysis**	**0.00136**
Glioblastoma	1.9609	**Type**	**0.00940**
Skull	1.8906	**New**	**0.00843**
Cause	1.8902	**Level**	**0.00694**
*⋯*	*⋯*	***⋯***	***⋯***

**Table 2:** Total generated frequent and infrequent itemsets using different support values.

Support	Frequent itemsets	Infrequent itemsets
0.1	146474	268879
0.15	105397	329766
0.2	94467	447614
0.25	79871	492108
0.3	57954	504320

**Table 3:** Positive and negative association rules using varying support and confidence values.

Support	Confidence	PARs from FIs	PARs from IIs	NARs from FIs	NARs from IIs
0.05	0.95	3993	254	27032	836
0. 05	0.9	4340	228	29348	1544
0.1	0.9	3731	196	30714	1279
0.1	0.85	3867	246	37832	1170
0.15	0.8	3340	330	26917	1121

**Table:** (a) Positive rules from frequent itemsets

Rule	Support	Confidence	Lift
{chemo radiation treatment} → {tumor}	0.2	1	2.0
{surgery tumor} → {radiation}	0.25	1	1.4286
{radiation surgery} → {tumor}	0.25	0.8333	1.6667
{treatment} → {radiation}	0.45	0.9	1.2857
{treatment tumor} → {radiation}	0.3	1	1.4286
{tumor} → {radiation}	0.5	1	1.4286

**Table:** (b) Negative rules from frequent itemsets

Rule	Support	Confidence	Lift
{radiation} → {~cancer}	0.5	0.7143	1.0204
{~temodar} → {radiation}	0.4825	0.8333	1.6667
{~radiation} → {glioblastoma}	0.65	1	1.4285

**Table:** (c) Positive rules from infrequent itemsets

Rule	Support	Confidence	Lift
{cancer treatment} → {tumor}	0.15	1	2
{doctor temodar} → {chemo}	0.05	1	2.8571
{brain chemo} → {doctor}	0.15	1	2.2222
{mri tumor} → {brain}	0.1	1	2.5

**Table:** (d) Negative rules from infrequent itemsets

Rule	Support	Confidence	Lift
{chemo} → {~surgery}	0.35	1	1.5385
{glioblastoma} → {~treatment}	0.3	1	2.13
{chemo} → {~glioblastoma}	0.35	0.95	1.4286
